# Activated coagulation time vs. Intrinsically activated modified rotational thromboelastometry in assessment of hemostatic disturbances and blood loss after protamine administration in elective cardiac surgery: analysis from the clinical trial (NCT01281397)

**DOI:** 10.1186/1749-8090-8-S1-P109

**Published:** 2013-09-11

**Authors:** M Petricevic, B Biocina, I Burcar, S Konosic, V Ivancan, R Gabelica, F Siric, T Kopjar, V Berkovic, H Gasparovic

**Affiliations:** 1 Department of Cardiac Surgery, University Hospital Center Zagreb, Zagreb, Croatia; 2 Department of Cardiac Anesthesiology, University Hospital Center Zagreb, Zagreb, Croatia

## Background

Excessive bleeding after cardiopulmonary bypass (CPB) is risk factor for adverse outcomes after elective cardiac surgery (ECS). Although many different point-of-care devices to diagnose hemostatic disturbances after CPB are available, the best test is still unclear. The study aim was to compare the accuracy of hemostatic disorder detection between two point-of-care devices.

## Methods

We enrolled 148 patients (105 male and 43 female) undergoing ECS in a prospective observational study. Rotational thromboelastometry (TEM, with InTEM test), and Activated coagulation time (ACT) measurement were performed 15 min after protamine administration. The cohort group was divided into two subgroups according to occurrence of excessive postoperative bleeding. Endpoints were defined in two ways: as total amount of chest tube output (CTO) and blood product transfusion requirements.

## Results

InTEM parameters, but not ACT, correlated significantly with CTO. Total amount of CTO value of 1507.50 mL presented 75th percentile of distribution, thus cut-off value for bleeder category. Patients in “Bleeder” category had longer CPB time (median, 116 vs. 95 min, p=0.023) and significantly lower value of the lowest body temperature during CPB (median, 30 vs. 32 degrees of Celsius, p=0.012). Patients with total amount of CTO exceeding 75th percentile were more frequently transfused with fresh frozen plasma (51.4% vs. 9.9%, p<0.001), fibrinogen concentrate (21.6% vs. 2.7%, p=0.001) and platelet concentrate (13.5% vs. 0.9%, p=0.004).

## Conclusion

Our study showed that InTEM test, but not ACT is useful for detection of hemostatic disorder after protamine administration following weaning from CPB. With aim to reduce excessive postoperative CTO, as well as excessive transfusion requirements, hemostatic interventions with timely and targeted blood component therapy according to InTEM results should be considered.

